# “Our work is recognized and we are prepared:” A qualitative evaluation of a peer-led research best practices training for community health workers and promotoras

**DOI:** 10.1017/cts.2025.10094

**Published:** 2025-07-21

**Authors:** Alexandra E. Harper, Analay Perez, M. Miaisha Mitchell, Daphne Watkins, Gina M. Jay, Vanessa I. Trujillo, Kristen Weeks-Norton, Shannen McIntosh, Brenda Eakin, Elias Samuels, Gretchen Piatt, Catherine Striley, Linda Cottler, Sergio Aguilar-Gaxiola, Susan L. Murphy

**Affiliations:** 1 Michigan Institute of Clinical and Health Research, University of Michigan, Ann Arbor, MI, USA; 2 Department of Family Medicine, University of Michigan, Ann Arbor, MI, USA; 3 Greater Frenchtown Area Governor’s Revitalization Council Inc, Tallahassee, FL, USA; 4 School of Social Work, University of Michigan, Ann Arbor, MI, USA; 5 Department of Physical Medicine and Rehabilitation, University of Michigan, Ann Arbor, MI, USA; 6 Clinical and Translational Science Center, University of California, Davis Health, Davis, CA, USA; 7 Center for Reducing Health Disparities, University of California, Davis Health, Davis, CA, USA; 8 Department of Anesthesiology, University of Michigan Medical School, University of Michigan, Ann Arbor, MI, USA; 9 Department of Learning Health Sciences, University of Michigan Medical School, University of Michigan, Ann Arbor, MI, USA; 10 Department of Epidemiology, College of Public Health and Health Professions and College of Medicine, University of Florida, Gainseville, FL, USA; 11 Department of Internal Medicine, Center for Reducing Health Disparities and Clinical and Translational Science Center, University of California, Davis Health, Davis, CA, USA

**Keywords:** Community health workers, promotoras, research training, research competency, qualitative research

## Abstract

**Introduction:**

To overcome the dearth of high-quality, evidence-based, role-specific training for community health workers and promotoras (CHW/Ps) working with research teams, we developed and evaluated a peer-led research best practices training for CHW/Ps. This article qualitatively explores the training experiences from the perspectives of CHW/Ps.

**Methods:**

CHW/Ps were recruited from communities and partners affiliated with study sites in Michigan, Florida, and California to participate in peer facilitated trainings in English and Spanish. A purposeful sample of CHW/Ps completed interviews from July to December 2023 about their perceptions of the training. Three coders analyzed the interviews using a combination of the rigorous and accelerated data reduction (RADaR) technique and thematic text analysis.

**Results:**

Seventeen CHW/Ps participated in interviews (*N* = 10 in English; *N* = 7 in Spanish). The mean age was 43.7 ± 14.5 years, most were female (59%), and 47% identified as Hispanic, Latino, or Spanish. We identified three primary themes, each with resulting subthemes: (1) CHW/Ps’ Perceptions of the Training, (2) Factors Influencing Receipt of the Training, and (3) CHW/Ps’ Recommendations for Future Trainings. Despite offering some opportunities for improvement in various areas, CHW/P learners found the training further clarified their role as CHW/Ps, enhanced their knowledge and skills, and provided a beneficial foundation for CHW/Ps working in community-engaged research.

**Conclusion:**

A peer-led research best practices training for CHW/Ps is an effective strategy for enhancing CHW/Ps’ knowledge and skills. Training CHW/Ps in research best practices is a strategy for enhancing the capacity and capability of this community-engaged research workforce

## Introduction

Community health workers and promotoras (CHW/Ps) engage in direct service provision, health education, advocacy, and community capacity building as well as help individuals navigate the healthcare system [1,2]. Moreover, their role in health-related research has been increasing as they are often perceived as trusted liaisons in underserved communities by amplifying the voices of the community’s needs, concerns, and perspectives about health research as well as highlighting pressing disparities that need attention [2–5]. While CHWs provide these services in the community, generally, promotoras are committed to providing culturally and linguistically relevant services to Spanish-speaking communities [6]. By engaging meaningfully with research teams, CHW/Ps can help bridge the significant gap between researchers and communities. For example, because the CHW/Ps often come from historically marginalized communities, researchers have increasingly integrated CHW/Ps in the research team to meaningfully engage these populations to augment representation and overcome limitations in understanding the unique socioeconomic and cultural factors affecting those populations [7,8]. Despite this critical role, the benefits of including CHW/Ps in research, and attempts to establish a set of core competencies [9], CHW/Ps often receive no standardized research training. While some state-level requirements for training and competencies exist, national competencies are not followed or do not [10–12]. As such, some CHW/Ps can be involved in research tasks beyond their acquired skillset, resulting in potential issues with scientific rigor, such as the inability to describe or reluctance to apply randomization principles and inadequate maintenance of participant confidentiality in community settings [13].

**Figure 1.**
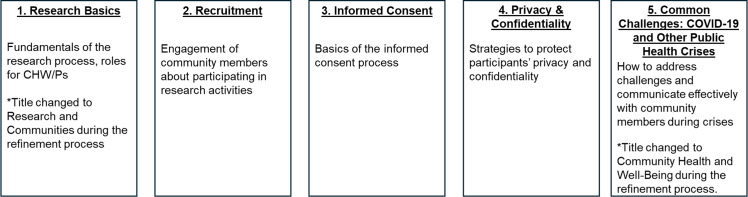
Five module research best practices training for community health workers and promotoras (CHW/Ps).

In response, our diverse team of researchers, CHW/Ps, and healthcare providers co-developed a culturally and linguistically tailored research best practices training for CHW/Ps in English and Spanish [14–16]. The training, which aimed to provide foundational education in role-specific best practices for research, consists of five modules (Figure 1) and can be accessed in several different formats. One way CHW/Ps can participate in the training is via a self-directed, asynchronous online format, which we previously found feasible and effective at increasing CHW/P learners’ perceptions of their knowledge and skills in participating in community-engaged research [15]. Alternatively, CHW/Ps can also participate in a synchronous peer-led “Champion” facilitated version of the training conducted virtually or in-person. A recent quantitative evaluation of the peer-led training found this format satisfactory and effective for increasing CHW/Ps’ perceived knowledge and skills; no meaningful differences were found in CHW/Ps’ ratings of perceived knowledge or skills based on site or modality (i.e., virtual or in-person) [16]. Given that no quantitative differences were found in modalities, the purpose of this qualitative study was to further explore the nuance in our findings and to identify opportunities to enhance the research best practices training for CHW/Ps.

## Materials and methods

We used a combination of criterion sampling and maximum variation sampling to identify CHW/P participants for this study [17]. We took this multi-pronged approach to sampling to conduct a representative qualitative evaluation of the training, seeking to capture perspectives from (a) CHW/Ps across sites who rated the training better or worse than the average of their peers at the site and (b) a diverse group of CHW/P learners. Using criterion-based sampling, we invited participants to the qualitative interviews based on site-level averages of training experience ratings. Specifically, we recruited equal proportions of CHW/P participants who scored above or below the average training experience for each site (California = 4.83 out of 5, Florida = 4.83 out of 5, Michigan = 4.66 out of 5, overall 4.77 out of 5). We also used maximum variation sampling to identify potential participants across four characteristics: gender, training language, past research experience, and training mode (e.g., virtual or in-person). Of the 394 CHW/Ps who completed the Champion facilitated training, 39 were invited to participate in an interview; 26 agreed and 17 participated, of whom 10 were English-speaking and 7 were Spanish-speaking. Nine invited individuals did not participate because they were unresponsive to repeated attempts at scheduling. This study was considered exempt by the University of Michigan Institutional Review Board, and all participants completed verbal informed consent with the interviewer.

Interviews were conducted with members of the research team in the preferred language of the CHW/P (i.e., English or Spanish). Interview protocols and guides were developed in both English and Spanish (see Supplemental Materials). The interview guide asked CHW/Ps’ role in the community, gauged their feedback about the training (e.g., likes, dislikes, suggestions for improvement, and modality of training) as well as strategies for disseminating the training to other CHW/Ps. All interviews were video- and audio-recorded via Zoom with participant consent. Interviews lasted approximately 30 minutes, ranging from 15 minutes for the shortest and 56 minutes for the longest. The team at University of California, Davis included Spanish-speaking individuals who facilitated the interviews in Spanish. Once the interviews were completed, they used a qualified bilingual translation editor for the initial transcription and translation then had bilingual team members review the translated transcripts for content and contextual accuracy to ensure that no nuance was lost. Analysis was conducted in English, but we cross-referenced anything that was unclear against the original Spanish transcript as needed to verify consistency and appropriateness. To begin data analysis, the two first authors used the rigorous and accelerated data reduction (RADaR) technique for the purposes of data reduction and to develop a codebook. The RADaR technique is a five-step qualitative data analysis method that encourages researchers to use word-processing software to organize, reduce, code, and analyze qualitative data [18]. Two authors (AP, AH) first organized and reduced the data that were not relevant to the research aims using Microsoft Excel. This included ensuring that the data transcripts across all participant interviews were formatted in the same way (step 1), reviewing the transcripts and noting similarities and differences across participant responses (step 2), and graying out redundant information that did not align with the research aims (steps 3 and 4). These steps were completed individually, then discussed between the two coders until consensus was achieved. After the data were reduced, the coders developed a codebook, and all transcripts were coded by the two coders and reviewed to mitigate coding discrepancies.

A Champion CHW/P (MM) also participated in the coding process to further enhance the community-engaged participatory research practices and help us to better understand and amplify the insider (i.e., CHW/P’s) perspectives. “Champions” are the individuals who facilitated the research best practices training and are CHW/Ps or otherwise work or volunteer in community-based organizations. The Champion CHW/P independently coded nine transcripts (53% of interviews) and met with the coders to discuss additions or discrepancies during the coding process. At the final stage, MAXQDA 2024 (Verbi, Germany) was used to facilitate the analysis process. We used thematic text analysis to generate categories and grouped similar categories into overarching themes [19]. The goal of thematic text analysis was to categorize the data to establish thematic categories or overarching themes that enable a deeper understanding of the data by identifying patterns or relationships. As such, we used an inductive, data-driven approach to generate the themes from the initial codes and categories [19,20].

## Results

As seen in Table 1, of the 17 interview participants, six attended a Spanish training led in California, one attended a Spanish training led in Florida, and 10 attended an English training in Florida, Michigan, or California; 12 attended the training virtually via Zoom. The average age of participants was 43.7 years ± 14.5, most were female (59%), and approximately half (48%) had prior experience as a member of a research team conducting community-engaged research.

**Figure 2.**
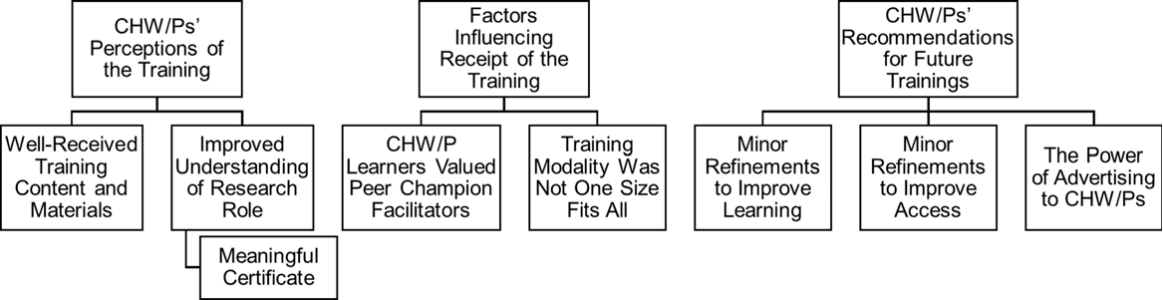
Themes and subthemes.

We identified three overarching themes from the qualitative analysis that each consisted of subthemes. The overarching themes identified were: 1) CHW/Ps’ Perceptions of the Training; 2) Factors Influencing Receipt of the Training; and 3) CHW/Ps’ Recommendations for Future Trainings (see Figure 2).

**Table 1.** Sample characteristics of participants (*N* = 17)

Characteristic	*N* (%)
Site	
California	7 (41.2)
Florida	5 (29.4)
Michigan	5 (29.4)
Ethnicity	
Hispanic/Latino/Spanish origin	8 (47.1)
Not Hispanic/Latino/Spanish origin	8 (47.1)
Declined to answer	1 (5.9)
Race	
Asian	1 (5.9)
Black/African American	5 (29.4)
White	8 (47.1)
Other race	1 (5.9)
Declined to answer	2 (11.8)
Gender	
Female	10 (58.8)
Male	7 (41.2)
Sex on Birth Certificate (Female)	10 (58.8)
Age, mean years (SD)	43.7 (14.5) range 20–65
Years Working as a CHW/P, mean (SD) Years Working as a CHW/P, median (IQR)	7.77 (8.3) range 0–28 6 (1.25, 11)
Education	
< High school	1 (5.9)
High school diploma or GED	2 (11.8)
Some college	3 (17.6)
Associate degree	5 (29.4)
Bachelor’s degree	4 (23.5)
Masters or Doctorate	2 (11.8)
Languages spoken by CHW/Ps	
English only	8 (47.1)
Spanish only	1 (5.8)
Bilingual (English and Spanish)	8 (47.1)
Professional Status, n (%)*	
Currently employed as a CHW/P	11 (64.7)
State certified as a CHW/P	6 (35.3)
Currently volunteer as a CHW/P	7 (41.2)
Experience as member of a research team conducting community-engaged research (Yes)	8 (47.1)
Training Modality	
In-person	5 (29.4)
Virtual (Zoom)	12 (70.6)
Training Language	
English	10 (58.8)
Spanish	7 (41.2)

Note: * = nonmutually exclusive category can exceed 100%; CHW/P = community health worker/promotora; GED = general educational development (diploma).

### CHW/Ps’ Perceptions of the Training

Participants discussed their varying perceptions of the training as learners, including two subthemes – *well-received training content and materials* and *improved understanding of research role.* Within the *improved understanding of research role* subtheme, a secondary subtheme arose relating to the *meaningful certificate* of completion. Brief descriptions and representative quotes for each subtheme are presented in Table 2.

**Table 2.** CHW/P learners’ perceptions of the training

Subtheme(s)	Description	Exemplar Quote(s)
Well-Received Training Content and Materials	General reactions to the training, including content and materials.	“Entonces creo que saber eso, que yo decía, pues eso es nomás para los investigadores o para la gente que es profesional, creo que es una cosa que cambio mi manera de pensar, y me gustó mucho. No puedo decir que no me gustó algo porque creo que todo estaba muy interesante.” [7, CA] “Knowing those facts that I thought were only for researchers or professionals is something that changed the way I think, and I liked it a lot…I can’t think of anything that I didn’t like because everything was very interesting.” [translation] “A lot of the training might have been a little repetitive from trainings I’ve received before, which is not necessarily a bad thing. It’s a good refresher…Having this program as like the initial training would be helpful for CHWs. Maybe making it mandatory or at least making it very appealing to others as a way to become CHWs.” [8, FL] “I liked the focus on promotoras as well as community health workers…I like the fact that a lot of the people in the video represented people that I grew up around and things like that. I also appreciated the overview of research basics…research basics are something that’s really important to go over…especially when you’re working with anything study related, understanding the informed consent and like what you can or cannot do is important…I liked how it was interactive, as well.” [11, FL] “I thought it was a necessary conversation…the energy was great.” [16, MI]
Improved Understanding of Research Role	Perspectives on how the training made participants feel about their role and ability to do their job.	“It helped me to understand, first of all, what a community health worker or promotora is. It also helped me to see how I fit in that role and what their purpose is…I saw myself understanding more of how [UC Davis’s] research program found itself in the community and how community health workers are important as trusted liaisons. It just reconfirmed some things for me about making sure that if you’re gonna be a liaison or ambassador in the community that trust is really important, especially among populations that look like you.” [1, CA] “Con lo que ustedes nos brindan, me están dando la oportunidad de desempeñarme mejor, más efectivamente…para mí es importante seguirme preparando para poder desempeñarme bien…Es como les digo, nuevamente somos los ojos en la comunidad y nos convertimos en las voces de la comunidad.” [2, CA] “With what you give us, you are giving me the opportunity to better perform my job, or more effectively…For me, it’s important to keep getting training to perform better…We are the eyes of the community, and we become the voices of the community.” [translation] “I get to know much about the work I’m doing, the job I’m doing, you know, and I’ve been putting it to use.” [9, FL] “¿Cómo se dice esa palabra? Importantes. Nosotros somos las personas que estamos ahí al frente que… somos las primeras como los First Responders… somos necesarios, somos personas que somos necesarias.” [12, FL] “How do you say that word? Important. We are the people that are on the front lines. We are the first ones, like the First Responders…it brought up that we are needed…[and] reminds us how important we are.” [translation] “[The training can] build your toolbelt…being more inform[ed] on different subjects [and] topics” [15, MI] “It’s empowering.” [16, MI]
Meaningful Certificate	Opinions about receiving a certificate of completion.	“Receiving the actual certification letter can demonstrate to my leadership that I was certified in this or I received the training; it’s a formal process. I think if someone, if I was a promotora and someone wanted to know my credentials, then having that certificate to show them that I went through some formalized training would be important for them to see that I’m not just saying these things from my own point of view; I had a trustworthy training to guide me through.” [1, CA] “[Demuestra] que nuestro trabajo se está valorando… Porque, esto no es solamente entre nosotros, entre los promotores que estamos aquí en esta comunidad, sino en todos los lugares que usted mencionó al principio, las universidades. Es decir, que se está reconociendo este valor que tenemos los promotores, que se nos está reconociendo nuestro trabajo.” [3, CA] “[It shows] that our work is being valued because this is not only among us, the promotoras who are in this community, but all the places, the universities. That is, the value that we have as promotoras is being recognized, that our work is being recognized.” [translation] “I felt, when I receive the email for the certificate, it had my name on it, so I felt seen, and I felt my presence was acknowledged. I felt appreciated, and I have something to show for like, ‘okay I attended the training, and it was this day.’ So, I was actually proud…it gives me a sense of belonging…” [10, FL] “For me, it’s just a certificate. We utilize those to convince academia. The community don’t care.” [16, MI]

#### Well-received training content and materials

CHW/P learners reported various broad reactions to the training; most reactions were positive and noted that the training content and materials were well received. Participants appreciated the opportunity to meet and interact with other CHW/Ps, enjoyed the video scenarios and knowledge checks embedded within the training, and thought the training was completed in an appropriate length of time (i.e., not too short or too long). Further, participants reported that the presentation of the content was inclusive, demonstrating diversity in pictures, videos, and scenarios, as well as representative of their work as CHW/Ps. Although two participants identified some content that felt repetitive to prior trainings (e.g., how to communicate with community members), everyone reported the training covered content and skills essential for CHW/Ps.

#### Improved understanding of research role

Along with reactions to the training, CHW/P learners discussed its effects on them as it related to their work. This subtheme refers to CHW/Ps’ perspectives on how the training made participants feel about their roles and ability to do their jobs. All participants reported some positive effects of the training. Specifically, the training clarified their role in research and helped CHW/Ps feel empowered and more confident, better prepared to engage in research-related activities, and valued as members of the community-engaged research workforce. Some participants reported that having access to this training demonstrated that those in academia valued their work and their role.

#### Meaningful certificate

Aside from the effects of the training, receipt of a certificate of completion emerged as a particularly important component for 14 of the 17 participants. The certificate demonstrates CHW/Ps’ competence and dedication to continued learning to their community and academic partners, can be an incentive to attend the training, and, pragmatically, helps track the trainings they have attended. Further, receiving the certificate was a source of pride. The remaining three participants had neutral perceptions of the certificate, noting that it was not necessary for them to obtain one, nor did it incentivize their attendance.

### Factors Influencing Receipt of the Training

Participants were keen to note factors influencing the receipt of the training from their perspective, resulting in two subthemes – the *CHW/P learners valued peer Champion facilitators* and *training modality was not one size fits all* (i.e., virtual vs. in-person). Brief descriptions and representative quotes for each subtheme are presented in Table 3.

**Table 3.** Factors influencing receipt of the training

Subtheme(s)	Description	Exemplar Quote(s)
CHW/P Learners Valued Peer Champion Facilitators	Perceptions of the role and traits of the Champions facilitating the training.	“Lo que me gustó es que todo era [la facilitadora] muy fácil de entender… y lo que más me gusto es que había ejemplos.” [6, CA] “What I liked is that [she] was very clear with all the concepts, but what I also liked was that there were examples.” [translation] “Yo creo que la persona o la facilitadora quería saber que las personas tenemos una sabiduría y tejer el entrenamiento con esa sabiduría de parte de todas. Siento que esa es una cosa que yo valoro mucho, que dan ese valor a la sabiduría que la gente ya tiene y ya posee… Me encantó que [la facilitadora] dio bastante espacio para el diálogo.” [7, CA] “I think that the person or facilitator wanted to know about the wisdom people have and intertwine it with the training. I feel that is something I value a lot. They provide that courage and the wisdom that people already have and possess… I loved the way she [the facilitator] gave us enough time to share.” [translation] “They gave us room to ask a question, explain ourselves without any form of discrimination or any interruption. So, I think the freedom of speech and the smoothness in the flow of how we conversate and how they explain things using examples we could understand…it makes me value that…They were really great and friendly. That made me enjoy the training due to their fluency and how they organized.” [10, FL] “The people facilitating it…were very well spoken, knew how to take, you know, what can be scientific jargon at times and turn it into plain language.” [13, MI] “The instructor was very informed in terms of how to get this information out. The instructors have to be able to instruct, so definitely she had it broken up very nice, everything.” [16, MI]
Training Modality Was Not One Size Fits All	Feedback about the training modality: in-person or virtual.	“I learn better in-person and interacting with people and that kind of dynamic, so I felt like that was a positive…If there’s a choice for it to be online, then of course because not everybody can make it in person…my preference is I think in-person has a lot more value.” [1, CA] “Yo creo que está bien porque uno está mucho más tranquilo en su casa o donde quiera que lo tenga. No tiene las prisas de que si voy a llegar tarde y tiene lo necesario aquí para empezar la capacitación. Creo que, me parece mucho más rápido por Zoom…Yo creo que es mucho más accessible…Es menos tiempo gastado en trasladarme a otro lugar. [6, CA] ‘I think it’s alright because you’re better off at home or wherever you are when you’re taking it. You don’t feel pressured about being late. You have everything that is necessary here to take the training… I think that Zoom training is much faster… I think that it was a lot more accessible…It’s less time that I would spend going to the location.’ [translation] “Pues virtual es muy conveniente, creo que poder ofrecer en persona y virtual es bueno. Creo que ayuda bastante. La manera, el aprendizaje obviamente en persona y en línea es diferente.” [7, CA] Well, it’s very convenient if it’s offered virtually. I think that it’s good if it can be offered in-person and virtually. I think that helps a lot. The way people learn online is different than the way people learn in-person.” [translation] “I don’t see any particular difference going in-person or being online.” [9, FL] “The first thing I loved about the training was the training being virtual. You know, it gives me the advantage of being able to attend the training from the comfort of my home and just through my computer.” [10, FL] “It was convenient…Zoom fatigue is settling on in. It’s weird…But because of how the modules were presented, nothing was lost in terms of value from it being, you know, on a computer screen or…Zoom.” [13, MI] “I do better when it’s not virtual. So I did appreciate that aspect. It was a little hard because it was held at a senior center. So there were some people like just coming in, and were just curious as to what was going on. It was also a mask requirement, so it could get a little uncomfortable just sitting there with the mask on, but again, I like things being in-person rather than being online, even though I know it’s easier when it’s online.” [14, MI]

#### CHW/P learners valued peer Champion facilitators

All participants reported neutral-to-positive feedback about the Champions facilitating the training and commented about the value of having peer facilitators. Participants noted key traits of effective Champions, such as being personable and communicating in a warm and engaging tone, as well as demonstrating active listening, patience, and professionalism (e.g., punctual, articulate in the language of learners). They also identified actions that Champions took to effectively facilitate the training, such as managing time (e.g., pacing of information, offering breaks), providing clarifying answers to questions, allowing space for participants to share experiences and engage with each other, and communicating scientific information in plain, easy-to-understand language.

#### Training modality was not one size fits all

Aside from gleaning information about whether it was important to have the facilitator be a peer, we also gathered substantial insights from participants about the environments in which they learned. Specifically, participants had mixed opinions about the training modality – virtual or in-person. Some participants preferred and noted the benefits of virtual training, including convenience and decreased burden to attend (e.g., fuel costs, travel time). Other participants preferred and noted the benefits of in-person training, such as the ease of interacting with other participants and it is how they are accustomed to learning.

### CHW/Ps’ Recommendations for Future Trainings

CHW/Ps offered several recommendations on ways to further enhance the current research best practices training for CHW/Ps. These recommendations are divided into three subthemes: *minor refinements to improve learning, minor refinements to improve access*, and *the power of advertising to CHW/Ps* (Table 4). *Minor refinements to improve learning* refer to ways the training could be refined by enhancing either the training content or material or increasing dedicated time for interactions. The subtheme *minor refinements to improve access* refers to potential logistical modifications to improve the training. The final subtheme, *the power of advertising to CHW/Ps*, refers to the increased need to advertise this training across different groups and use various suggested strategies by CWH/Ps.

**Table 4.** CHW/Ps’ recommendations for trainings

Subtheme(s)	Description	Exemplar Quote(s)
Minor Refinements to Improve Learning	Suggestions for opportunities to improve training content and materials to promote learning.	“No sé si los subtítulos eran en inglés o no todo el video era en español. Nos hubiera agradado que todo hubiera sido 100% en español…Si hablan en español, también los subtítulos en español para que la persona pueda entender bien. Hay unos que no pueden leer bien en español.” [5, CA] “I don’t know if subtitles were in English or the whole video wasn’t in Spanish. We would have liked it if 100% of it was in Spanish…So if they speak Spanish [in the video], the subtitles need to be in Spanish so the person understands well. There are some that can’t read well in Spanish.” [translation] “I feel like the discussion prompts were for people who had experience as a community health worker.” [11, FL] “If I could have reading material, that helps, too, so I can look back and refresh because sometimes like your auditory processing and your visual processing need to work together. Because if I’m just hearing, I’m not, sometimes I don’t get it. But to be able to look back afterwards and soak it in, that would have helped a lot. I love a good workbook that I can like take notes in the margin and that has like easy-to-read font.” [14, MI]
Minor Refinements to Improve Access	Insights on modifying logistical components of the training to improve the training experience.	“Tal vez que dieran como opciones de horas, porque tal vez no me tocó en las mañanas porque trabajo en las mañanas y no quedar una hora tan, o sea no se decir las siete y las que pueden la mañana, pues la mañana. O el fin de semana, aunque el fin de semana si no es en persona es muy difícil que las personas se unan online, si no es en persona.” [4, CA] “You could provide options regarding the time because maybe not in the morning because there’s work in the morning, but sometime in the afternoon And those who can do it in the morning can go in the morning or during the weekend. However, during the weekend, if it’s not in-person, it’s very difficult for people to get together online if it’s not in-person.” [translation] “My biggest modification would be to try as much as possible to maybe do it in-person. To also kind of foster the conversation a little bit better because I know that a lot of people are used to using Zoom and things like that, but I feel like, especially if you’re like doing it within an organization and with coworkers, it may be best to have someone like go in-person and present it.” [11, FL] “I need a lot of breaks. I need to be able to stand up, stretch, move… So having more breaks in the middle would have been beneficial to me.” [14, MI] “I think that overtime, people kind of long for a more intimate engagement. So maybe a hybrid kind of thing.” [17, MI]
The Power of Advertising to CHW/Ps	Strategies for advertising the training to enhance participation.	“Have someone that is a community leader go through the training…city council person or assembly person that represents that district or represents that zip code and where people live, maybe a civic leader. They go through the training, and then they go out and talk to the people. Of course, I think faith-based organizations could help, as well. So if you have a faith-based leader or faith-based ambassador who believes in this type of work, then…they could be a liaison to the community that they serve.” [1, CA] “Creating a flyer…you put it out there on social media. And also let them know that…people of all ethnic groups are welcomed…because sometimes when people saw things like this…probably someone from the Hispanic group…Asian group…or Black community, they tend to feel reluctant to attend or probably to join… This is actually a sign or…a phrase telling them, signifying to them that, okay, they are welcome. They are welcome to come there and share their opinions.” [9, FL] “You could advertise is on, you know, social media as well, just for broader network. If you work somewhere where there’s multiple CHWs or even CHW-adjacent professionals, just suggest it, put it in an email chain…There’s a national CHW coalition, various social services organizations that we’re familiar with.” [13, MI]

#### Minor refinements to improve learning

Participants discussed making potential modifications to the training content. For example, participants mentioned that the module on COVID was not as relevant as it was a few years ago. Some participants also suggested adding a section on statistics within a public health or community health context, such as learning about chronic conditions within a specific state or city and having more conversations around implicit bias and the health needs of particular groups (e.g., African Americans). Participants also vocalized the importance of obtaining access to the PowerPoint slides used in the training. One participant noted that although they wrote notes during the training, they were often unable to write all the information presented to them. Another participant shared the idea of offering a workbook that would allow them to take notes on paper and have relevant training information. Therefore, having a set of slides would ensure they have more complete information. Participants also recommended that a glossary of concepts be included as part of the training, especially if words are not familiar to participants (e.g., randomization), and the language for the training videos and subtitles be consistent. Participants also suggested increasing opportunities throughout the training for peer interaction in groups to learn from each other and their experiences in the field.

#### Minor refinements to improve access

Participants offered several considerations regarding the training to improve their ability to access and attend the training. These included suggestions for modifications to logistical and structural components of the training. Participants discussed offering multiple in-person options that are centrally located in the community. Given that a virtual option was offered, participants acknowledged that while this is convenient for many, everyone has differing knowledge of and access to various types of technology. Thus, they suggested including a survey question when registering for the training to determine people’s level of comfort with technology and including a video to demonstrate how the online platform would look for participants taking the training. Participants also suggested giving the option of a flexible schedule and offering the training multiple times, such as early morning, late evening, or on weekends. Regarding the training structure, one recommendation offered was to embrace a “train the trainer” model that enables CHW/P attendees to become trainers to lead this training in the future. Overall, participants shared the benefits of an in-person option with increased face-to-face interaction and acknowledged the convenience of online training, especially when weather could present challenges.

#### The power of advertising to CHW/Ps

Participants discussed three different avenues for advertising the research training for CHW/Ps. These were categorized into 1) among CHW/Ps themselves, 2) the communities in which CHW/Ps live, and 3) across media outlets. Participants expressed sharing information about the training among themselves across organizations where they work, such as local, state-based, or national CHW organizations, organizational websites specific to CHW/Ps (e.g., The National Association of Community Health Workers – https://www.nachw.org), and nonprofit organizations. They also recommended partnering with CHW/P-specific accounts on LinkedIn and social media. Several community spaces where CHW/Ps live were also discussed as potential areas to disseminate information about the training to reach other CHW/Ps. These locations include a religious assembly hall or church, barbershops, beauty salons, public health centers, clinics, schools, housing commissions, legal clinics, and mental health organizations. Participants also discussed the widespread use of social media and suggested sharing information about the training across social media, including Facebook, Instagram, and TikTok to “reach a broader network” (13) more efficiently. Overall, participants acknowledged the importance of disseminating information about the training with CHW/Ps and using multiple modalities across different avenues.

## Discussion

As a strategy to enhance CHW/Ps’ ability to be effective members of community-engaged research teams and to address a gap in this vital workforce’s knowledge and skills, we developed a research best practices training for CHW/Ps. Regardless of whether participants completed the English or Spanish training in-person or virtually, CHW/Ps reported positive feedback about the training. Existing literature provides mixed findings on the benefits and drawbacks of in-person and virtual training [21–24]. Our results indicate that the choice of training modality varies and depends on the preferences and priorities of the staff and CHW/P learners at each site. Offering the choice of training modality appears important for meeting learners’ needs and building capacity for CHW/Ps to engage in research. Our qualitative evaluation builds on the positive results from our quantitative evaluation [16], specifically revealing that CHW/Ps appreciated the training content, the flexibility to attend the training based on their preferences, and the academic community is recognizing the importance of CHW/Ps in conducting community-engaged research. Nonetheless, they reported opportunities for refining the training content and promoting its dissemination. These recommendations were practical and implementable to refine this already well-received training.

A few CHW/P learners reported that some content felt repetitive to other foundational trainings they had previously attended. Indeed, this likely reflects the variation in years of experience among our sample of CHW/Ps. However, this repetition seemed necessary as our training puts the information within the context of a research study. This shift in mindset required CHW/Ps to think about engaging community members to support health and well-being to think about engaging community members as research participants, thus requiring a different type of interaction and increased levels of scrutiny and protection. Data protection is critical when involving underserved and underrepresented communities in research given historical mistrust [25]. There is also value in repeatedly exposing learners to critical information to facilitate retention [26–28]. Further, our peer-led training offers the opportunity to interact with other CHW/Ps, which can be a beneficial strategy for promoting a deeper understanding among CHW/Ps and the communities they serve.

Our key takeaways for future iterations of the training include ensuring language congruence (i.e., all videos, subtitles, and handouts match the language of the training); maintaining cultural and linguistic representativeness in videos, images, and scenarios; and adding deliverables to support learning, such as a glossary of terms, workbook, and handouts of the slides. Further, learners perceived neither modality to be superior to the other, so we will continue to support sites by offering both in-person and virtual training based on the needs of their communities. Taken together, the results from our quantitative and qualitative evaluations demonstrate that the training is promising but needs minor refinements before undertaking scaling efforts.

As noted previously, there is limited and variable training in research best practices for CHW/Ps; some research teams provide study-specific training, but no standardized training exists that is accessible to all CHW/Ps. As such, our development and evaluation of a standardized research best practices training contributes to the limited research on role-specific training programs for CHW/Ps engaged in conducting research with communities. Given the challenges in recruiting and retaining participants of ethnic and racial minority groups across health-related studies, CHW/Ps can also help to bridge historically marginalized groups into research through the skills fostered by the research best practices training [29,30]. The research best practices training equips CHW/Ps with the necessary skills to recruit and inform potential participants about the research process, which can ultimately help to increase the inclusion of racial and ethnic minorities in research. It is important to note several differences pertaining to state requirements for CHW/Ps. Certification is recommended, though not required in Florida, is under development in California, and in Michigan, CHW/Ps require training, but no certification is necessary [31]. As a result, our sample represented varying scenarios as it pertained to state-regulated requirements. Importantly, regardless of state-specific regulatory requirements, CHW/Ps in each state found the training provided a critical foundation for understanding their role as it pertains to research. A future study could explore differences in training uptake across CHW/Ps living in states with different requirements to examine their receptivity to research best practices training. Ultimately, teaching CHW/Ps fundamental research skills can help them promote health equity and improve patient engagement and health outcomes [32].

### Limitations

One limitation was that most Spanish-speaking participants were from the California site, as the Florida and Michigan sites had less success recruiting Spanish-speaking participants for the original quantitative evaluation [16]. Further, we did not record the organizations where participants are currently employed as it was beyond the scope of this study. As a result, this could limit our understanding of the populations they serve and standard practices in their organizations. Nevertheless, our sample was diverse, and participants were well-represented across ethnicity, race, gender, education, and professional status. Another limitation is that the Champion CHW/P was able to code only half of the transcripts (53%). We decided to not have them code 100% of transcripts intentionally to preserve the community-academic relationship, acknowledging their own commitments and responsibilities as a highly involved member of the community. In this way, we attempted to balance gaining insights into the data with the time burden. CHW/P participants expressed their desire to have access to the PowerPoint slides used in the training. Due to ongoing development, we decided not to share the slides during the evaluation study; however, we plan to share the materials with CHW/Ps participants now that they are finalized. Lastly, we followed translation procedures recommended by the World Health Organization [33] and believe the steps our team of bilingual analysts took to ensure contextual accuracy, such as multiple reviews of the Spanish-to-English translation and cross-referencing the original Spanish transcripts for further clarity, prevented the loss of critical meaning or nuance during translation. It is possible that not conducting the analysis in Spanish resulted in some loss of nuance. However, we embraced a community-engaged approach to qualitative data analysis, requiring us to have the transcripts in English to engage our community partner, which we consider a strength of our methods [34]. Teams conducting qualitative analyses in languages other than English may consider different approaches.

### Future directions

We have finalized refinements and updates to the training based on feedback from our collaborators and CHW/P learners from this evaluation, such as providing handouts and ensuring language consistency. In this process, we leveraged the expertise of an instructional designer to maximize the accessibility and utility of the training and supporting materials. We are currently piloting the implementation of the refined training with four new sites with community-academic partnerships engaging CHW/Ps in their community-engaged research. This implementation will be focused on understanding the feasibility of and barriers and facilitators to scaling this training in preparation for a larger multisite hybrid effectiveness-implementation trial, a design well-suited to translating and scaling trainings into real-world settings [35].

## Conclusion

CHW/Ps expressed appreciation that the importance of their work was recognized and that they were more prepared to engage community members in the research process after attending the research best practices training for CHW/Ps, regardless of whether they were participating in the English or Spanish version of the training. Our team members, who were from diverse cultural and professional backgrounds, created this training to enhance the capacity of the community-engaged research workforce by providing standardized training for CHW/Ps. The refined English and Spanish training and materials are now publicly available at https://www.chwresearchtraining.org/.

## Supporting information

10.1017/cts.2025.10094.sm001 Harper et al. supplementary material Harper et al. supplementary material

## Supplementary material

The supplementary material for this article can be found at https://doi.org/10.1017/cts.2025.10094.
