# Supplementary material for: “Our work is recognized and we are prepared:” A qualitative evaluation of a peer-led research best practices training for community health workers and promotoras
Source: J Clin Transl Sci. 2025 Jul 21;9(1):e180. doi: 10.1017/cts.2025.10094 (PMC12444694; doi:10.1017/cts.2025.10094)
Supplement: Harper et al. supplementary material [file S2059866125100940sup001.docx]

Supplemental Material: English Interview Guide

*To begin, we would like to ask about your work as a CHW/P.*

Tell us a bit about your work as a CHW/P.

- What communities have you worked with?

1. Can you talk about what you liked and didn’t like about the training?
   1. Probes (optional to ask about): training experience, content, group format
2. What sorts of changes or modifications to the training do you think would improve this training and make it more likely for other CHW/Ps to attend?
3. What kind of barriers did you experience or do you think other CHW/Ps might have?
   1. Probes: access, connectivity, length of training
4. How important for you was it to receive a certificate of completion for attending training?
   1. Probes: What effect does this certificate of completion have on your job?

[For those who attended **Virtual Training**], Let’s switch gears and think about the virtual training.

1. In what ways was having the training conducted virtually over Zoom helpful or not helpful to your learning?
   1. Probes: scheduling/attendance

**Closing**

1. What are some ways you can think of for sharing this training with other CHW/Ps?
2. Can you speak about what it would look like for the community-based groups you’ve worked with to offer this training to other CHW/Ps?
   1. Probes: What would be helpful to facilitate this training for the community-based groups you’ve worked with? What would be difficult?
3. Is there anything else you want to mention about the training that we have not yet talked about?

*Thank you very much for your time and assistance*.

Supplemental Material: Spanish Interview Guide

*Para empezar, nos gustaría preguntarle sobre su trabajo como trabajadora de la salud comunitaria.*

Cuéntenos un poco sobre su trabajo como trabajadora de la salud comunitaria o promotora.

○ ¿Con qué comunidades ha trabajado?

1. ¿Puede hablarnos de lo que le gustó y lo que no le gustó del entrenamiento?
   1. Investigue: (es opcional preguntar sobre estos puntos): cómo le fue en el entrenamiento, contenido, formato de grupo.
2. ¿Qué tipo de cambios o modificaciones al entrenamiento cree que lo mejorarían y harían más probable que otras trabajadoras comunitarias/promotoras participaran?
3. ¿Qué tipo de barreras enfrentó o cree que podrían enfrentar otras trabajadoras de la salud comunitaria o promotoras?
   1. Investigue: acceso, conectividad, duración del entrenamiento.
4. ¿Qué tan importante fue para usted recibir un certificado de participación en el entrenamiento?
   1. Investigue: ¿Qué efecto tiene este certificado en su trabajo?

[Para quienes asistieron a **la formación virtual**]: Cambiemos de tema y pensemos en el entrenamiento virtual.

1. ¿De qué manera le ayudó o no le ayudó a aprender el hecho de que el entrenamiento se impartiera virtualmente a través de Zoom?
   1. Investigue: horario/asistencia

**Cierre**

1. ¿De qué otra manera piensa usted que se pudiera compartir este entrenamiento con otras trabajadoras de la salud comunitaria/promotoras?
2. ¿Puede comentar qué pasaría si los grupos comunitarios con los que ha trabajado ofrecieran este entrenamiento a otras trabajadoras de la salud comunitaria/promotoras?
   1. Investigue: ¿Qué sería útil para facilitar este entrenamiento a los grupos comunitarios con los que usted ha trabajado? ¿Qué sería difícil?
3. ¿Hay algo más que quiera mencionar sobre el entrenamiento que aún no hayamos comentado?

*Muchas gracias por su tiempo y por su ayuda.*
